# COVID-19 in Ghana: challenges and countermeasures for maternal health service delivery in public health facilities

**DOI:** 10.1186/s12978-021-01198-5

**Published:** 2021-07-19

**Authors:** Faith Agbozo, Albrecht Jahn

**Affiliations:** 1 Department of Family and Community Health, School of Public Health, University of Health and Allied Sciences, PMB 31, Ho, Ghana; 2 Heidelberg Institute of Global Health, Heidelberg University Medical Faculty, Im Neuenheimer Feld 130.3, 69120 Heidelberg, Germany

**Keywords:** COVID-19, Pandemics, Maternal Health, Health Services, Public Health, Developing Countries, Ghana

## Abstract

We provide a situational update on COVID-19 in Ghana, the seventh African country reporting the most cases. Some modifications occurring within the health system to curtail the outbreak and its potential impact on the delivery of antenatal care services are also highlighted. With the discovery of the Delta variant in Ghana, the current attention is to prevent a third wave of infection, and also control and manage existing cases. Efforts to procure vaccines, vaccinate special populations and sensitize the public on the implications of vaccine hesitancy are ongoing. Amidst these activities, we suggest some innovations and countermeasures to safeguard primary healthcare services and potentially reinvest efforts towards achieving the sustainable development goal three within the context of maternal healthcare, citing examples specific to developing countries.

## Background

Ever since the first case of the novel coronavirus (SARS-CoV-2) was notified in Ghana on March 12 2020, a total of 95,476 laboratory-confirmed infections have been reported from 1,264,190 samples tested, giving a daily confirmed case of 3,011 per million population as of June 29, 2021 [1]. Ghana has since moved from the third to the seventh position regarding covid-19 caseload in Africa with South Africa, Ethiopia, Kenya, Nigeria, Zambia and Algeria leading. Cumulatively, Africa regional case count comprises 3,942,448 infections and 94,217 deaths from 47 reported countries [2]. Despite the 9.6 positivity rate in Ghana, the number of deaths (795) and fatality rate (0.8%) are low due to the high recovery rate (97.7%) [1]. Although Ghana is touted as one of the successful countries in the sub-region for vaccine acceptance, having administered 1,232,876 vaccine doses to its 30.42 million estimated population until June 29, 2021, rollout of the second jab and expansion of vaccination to the general population is marred by procurement and supply difficulties [1].

In response to the pandemic, guidelines on case management of covid-19, antigen testing, and emergency preparedness and response developed by the Ministry of Health (MoH) with support from the Ghana Health and the World Health Organization have guided the prevention, control and management of the infection in Ghana [3–5]. To minimize infections in healthcare settings, the MoH recommended that healthcare workers apply standard and transmission-based infection prevention precautions consistently to all patients—symptomatic and asymptomatic alike—across all levels of healthcare [4]. Concerning health systems restructuring, the health ministry has directed medical care across all levels to make significant adaptations to routine procedures, the foremost being heightened hand and respiratory hygiene, triaging and source control, use of appropriate personal protective equipment, safe injection practices, decontamination of the environment and equipment, and safe corpse handling [4].

Reports from Annual Regional Health Review meetings and observations made during monitoring and supervisions show that at the point of entry to a hospital, body temperature is checked, and suspected cases are sent to designated isolation areas. Clients are cautioned to limit hospital visits unless extremely necessary. Specialist appointments have reduced, and non-essential medical procedures, including non-emergency surgeries, are less prioritized. Some community health professionals are reassigned to provide covid-19 related services, thereby limiting home visitations and other community-oriented activities. Due to variability in the health infrastructure, geographic location, and the range of services across primary, secondary and tertiary levels, district health management teams and individual facilities are responsible for deciding on the specific nature and extent of the adaptations.

## Main text

Already, maternal mortality in Sub-Saharan Africa and Ghana remains unacceptably high at 546 and 319 deaths per 100,000 live births, respectively [6]. This trend has largely been attributed to poor care-seeking behaviours, as 30% of pregnant women in Ghana do not seek antenatal care (ANC) in a health facility nor meet the target of at least four ANC consultations during pregnancy [7]. Covid-19 is feared to disrupt health services and programmes and deepen the already weak health infrastructure in many low-income and middle-income countries (LMICs) [8]. A case is ANC, which offers a unique opportunity to render a wide range of vital interventions comprising health promotion, disease prevention, screening, diagnosis and management of diseases.

Most public health facilities have made positive adaptations of expanding ANC waiting areas and allocating more days for ANC as a way of decongestion. Pregnant women are encouraged to make their first ANC booking in the second instead of the first trimester. Among non-high-risk women in their first and second trimesters, appointments are rescheduled to alternate monthly visits. However, high-risk pregnancies follow the standard ANC practice of eight ANC visits during pregnancy where there are no complications—one visit in the first trimester, two visits in the second trimester and five visits in the third trimester [9]. For complication-free deliveries, two-week postnatal visits are delayed until six weeks. Other adaptations intended to reduce close contact with the client include using handheld fetal doppler instead of the traditional Pinard fetoscopes to assess fetal heart rate. These changes are not peculiar to Ghana [8]. However, the setup of most delivery wards remains unchanged, and covid-19 infection by itself is not an indication for caesarean section.

Evidence suggests that these modifications are adversely influencing women’s experience of a positive pregnancy, patient-centred and respectful maternity care, as well as the coverage and regularity of ANC, postnatal and reproductive health visits [8]. Per projections, reductions in maternal health coverage by 9.8–18.5% during 6 months of covid-19 pandemic would result in additional 12,200 maternal deaths and 253,500 child deaths in LMICs [10]. Data from the District Health Management Information System (DHMIS) during January and May 2020 for the largest Ghana Health Service referral facility serving the epi-region showed a 25–65% decline in maternity service usage. Comparing the same period, ANC attendance (1697 vs 796), ANC registrations (167 vs 147), and haemoglobin checked at 36 gestational weeks (349 vs 225) dropped. Also, comparing May 2019 with May 2020, the indicators followed a similar decrease in ANC attendance (1434 vs 796), ANC registrations (199 vs 147), first-trimester registration (110 vs 78), haemoglobin checked at ANC registration (199 vs 147), and haemoglobin checked at 36 weeks (495 vs 225). If this decline continues, gains achieved in many maternal health targets will be wiped out.

To maintain service delivery, facilities mainly in the urban areas have explored telemedicine, but that has not gone beyond using voice calls and text messaging to reinforce health education and send reminders on appointments. Risk communication activities for maternal healthcare are established. These cover community engagement; development of broadcast materials; surveillance, home visits and contact tracing at the district, sub-district and community levels; support through call centers and community health officers; in addition to mass communication, outreach services and sensitization [3].

**Fig. 1:**
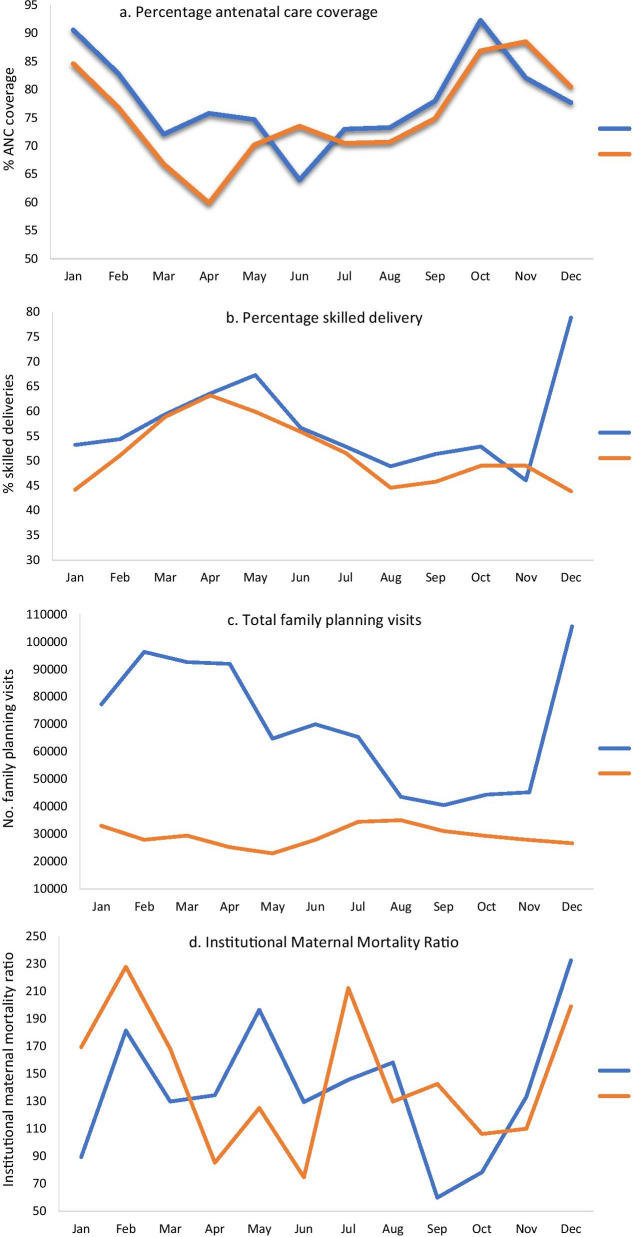
Comparison of **a** antenatal care, **b** skilled deliveries, **c** total family planning visits and **d** institutional maternal mortality ratio for the period of 2019 and 2020

In a country having 9293 public health facilities staffed by 88,205 health professionals [3], a key strength is the DHMIS data which enables the design of disease a robust surveillance information systems. Data from the DHMIS generally shows a downward trend in maternal health indicators between 2019 and 2020 in the region with the highest covid-19 case count (Fig. 1a–c). The consequence is the poor progress in decreasing fluctuations in institutional maternal mortality ratio (Fig. 1d). However, these health outcomes might be decreasing due to the restrictions in ANC visits, women’s apprehension of visiting health centres and probably reduced contact with clients due to health workers’ panic of being infected. In fact, 4763 out of the 95,476 (5.05%) covid-19 infections are among health workers with some casualties [2]. Nonetheless, infection rate among females is relatively lower in Ghana (42%) [1].

The WHO Regional Office for Africa alerts of a rising risk of covid-19 resurgence in Africa [2]. Between April and June 2021, six Delta variants (highly contagious strain) was detected at Ghana’s ports of entry. Fear of a third wave is drifting attention from routine clinical practice, necessitating innovative actions to safeguard maternal health services. Crucial is to release real-time epidemiologic data on the incidence, treatment outcomes and case fatality among pregnant women concurrent with COVID-19 update release for the general population. This would help assess constraints hindering maternal health outcomes, evaluate impact of control measures, and stimulate stakeholders’ involvement and governments’ commitment towards supporting maternal health interventions. Among vulnerable populations, it is helpful to enhance surveillance and collate covid-19 related sentinel data. In this regard, using maternal epidemiologic data from the DHMIS for timely public health decision making will facilitate planning and health systems modifications based on estimates. Furthermore, getting women and health workers’ feedback on the quality of maternal service received during the pandemic, its effect, and the rationale for reduced service utilization will direct the focus of guidelines and interventions tackling emergency preparedness. Outreach services, telemedicine, community engagement and information sharing could retain maternal health service provision during future health emergencies.

## Conclusion

Irrespective of outbreaks, governments in LMICs need to continuously invest in maternal health infrastructure as the implications span generations. The present covid-19 situation necessitates accurate and accessible information and access to comprehensive and quality obstetric support, including ensuring that all cesarean sections are medically justified. To promote attainment of the sustainable development goals, we propose an equitable allocation of health workers performing public health activities not deemed essential at all healthcare levels in all geographical zones. Also, health workers need to be equitably allocated to respond to covid-related illness in hard-hit areas and others to deliver routine care. Focused ANC should be integrated for women with multi-morbidities and chronic conditions, and health workers realigned to render services for high-risk and vulnerable women. Maintaining the continuum of care for maternal health services require community engagement and risk communication. Emphasis should be on stakeholder involvement in designing mitigation measures, adopting m-health technologies for patient teaching and consultations and applying lessons learnt to contain future health emergencies.

## Data Availability

All the data used have been reported in the manuscript.

## Abbreviations

ANC: Antenatal Care
COVID-19: Coronavirus 2019
LMIC: Low-Income and Middle-Income Countries
SARS-CoV-2: Severe Acute Respiratory Syndrome Coronavirus 2
